# Styryl-Based and Tricyclic Compounds as Potential Anti-Prion Agents

**DOI:** 10.1371/journal.pone.0024844

**Published:** 2011-09-13

**Authors:** Erika Chung, Frances Prelli, Stephen Dealler, Woo Sirl Lee, Young-Tae Chang, Thomas Wisniewski

**Affiliations:** 1 Department of Neurology, New York University School of Medicine, New York, New York, United States of America; 2 Microsens Biotechnology Ltd, Camden, United Kingdom; 3 Department of Chemistry & MedChem Program of Life Sciences Institute, National University of Singapore, Singapore, Singapore; 4 Laboratory of Bioimaging Probe Development, Singapore Bioimaging Consortium, Agency for Science, Technology and Research (A*STAR), Singapore, Singapore; 5 Department of Pathology, New York University School of Medicine, New York, New York, United States of America; 6 Department of Psychiatry, New York University School of Medicine, New York, New York, United States of America; INSERM, UMR-S747, France

## Abstract

Prion diseases currently have no effective therapy. These illnesses affect both animal and human populations, and are characterized by the conformational change of a normal self protein PrP^C^ (C for cellular) to a pathological and infectious conformer, PrP^Sc^ (Sc for scrapie). We used a well characterized tissue culture model of prion infection, where mouse neuroblastoma cells (N2a) were infected with 22L PrP^Sc^, to screen compounds for anti-prion activity. In a prior study we designed a library of styryl based, potential imaging compounds which were selected for high affinity binding to Alzheimer's disease β-amyloid plaques and good blood-brain barrier permeability. In the current study we screened this library for activity in the N2a/22L tissue culture system. We also tested the anti-prion activity of two clinically used drugs, trimipramine and fluphenazine, in the N2a/22L system. These were selected based on their structural similarity to quinacrine, which was previously reported to have anti-prion activity. All the compounds were also screened for toxicity in tissue culture and their ability to disaggregate amyloid fibrils composed of PrP and β-amyloid synthetic peptides in vitro. Two of the imaging agents, 23I and 59, were found to be both effective at inhibiting prion infection in N2a/22L tissue culture and to be non-toxic. These two compounds, as well as trimipramine and fluphenazine were evaluated in vivo using wild-type CD-1 mice infected peripherally with 139A PrP^Sc^. All four agents significantly prolonged the asymptomatic incubation period of prion infection (p<0.0001 log-rank test), as well as significantly reducing the degree of spongiform change, astrocytosis and PrP^Sc^ levels in the brains of treated mice. These four compounds can be considered, with further development, as candidates for prion therapy.

## Introduction

Prion diseases are conformational neurodegenerative disorders characterized by the structural modification of the normal prion protein, PrP^C^(C for cellular), into a pathological conformer, PrP^Sc^ (Sc for scrapie) [1], [2]. They are a unique category of illness in that they can be inherited, infectious or sporadic in occurrence. The human forms of prionoses include kuru, Creutzfeldt-Jakob disease (CJD), Gerstmann-Sträussler-Scheinker disease (GSS), fatal familial insomnia, sporadic fatal insomnia and the more recently described “protease-sensitive prionopathy” [3]–[5]. In animals these diseases include bovine spongiform encephalopathy (BSE) in cattle, scrapie in sheep and goats, chronic wasting disease (CWD) in deer and elk and transmissible mink encephalopathy (TME) [3], [4]. Currently there is no effective therapy for this group of diseases [2], [6]–[9]. The outbreak of bovine spongiform encephalopathy (BSE) and the resulting emergence of a new human prion disease vCJD, highlight the public health threat from prion diseases. In North America an ongoing threat from prion disease is from chronic wasting disease (CWD). High rates of infection among deer and elk populations have been reported, with experimental data indicating that this disease is transmissible to primates [10]–[12]. Hence there is a great need for effective therapeutic approaches for prion disease.

Numerous studies have tried to develop anti-prion therapies, typically utilizing prion infected cell lines where agents that decrease PrP^Sc^ levels can be identified. These have included diverse agents such as quinacrine, chlorpromazine, pentosan polysulfate, Congo red, tetracycline and dendritic polyamines [6], [8]. Various immunotherapeutic approaches have also been attempted [2]. Very few of these have been shown to be effective in vivo using animal models of prion scrapie infection and none have been shown to have a clinical effect in humans [6], [8]. A number of compounds which are amyloidophilic, such as Congo red derivates, have been shown to have some anti-prion effectiveness [13]–[16]. In addition compounds with a similar scaffold structure to quinacrine and chlorpromazine have been shown to have effectiveness in prion tissue culture [17], [18]. Important properties for potential anti-prion agents with in vivo effectiveness include the abilities to inhibit the prion replication in tissue culture, block the PrP^C^ to PrP^Sc^ interaction and to have high blood brain barrier (BBB) permeability [8]. In prior studies we designed a library of styryl based compounds which were selected for high affinity binding to β-amyloid plaques and likely good BBB permeability by virtue of having no charged moieties [19]. In the present study we screened this library for inhibition of prion infection using a N2a/22L tissue culture model [20], [21]. In addition we screened the effectiveness of fluphenazine and trimipramine, which are a phenothiazine and a tricyclic antidepressant, respectively, that are structurally similar to other anti-prion agents such as quinacrine and are known to be BBB permeable, as well as, being well tolerated in patients. All compounds with effectiveness in N2a/22L prion infection were screened for toxicity and their ability to inhibit both PrP and Aβ fibril formation in vitro. Effective and non-toxic compounds were then tested in vivo using CD-1 mice infected with 139A PrP^Sc^.

## Results

### Treatment of N2a/22L cells with compounds

**Figure 1:**
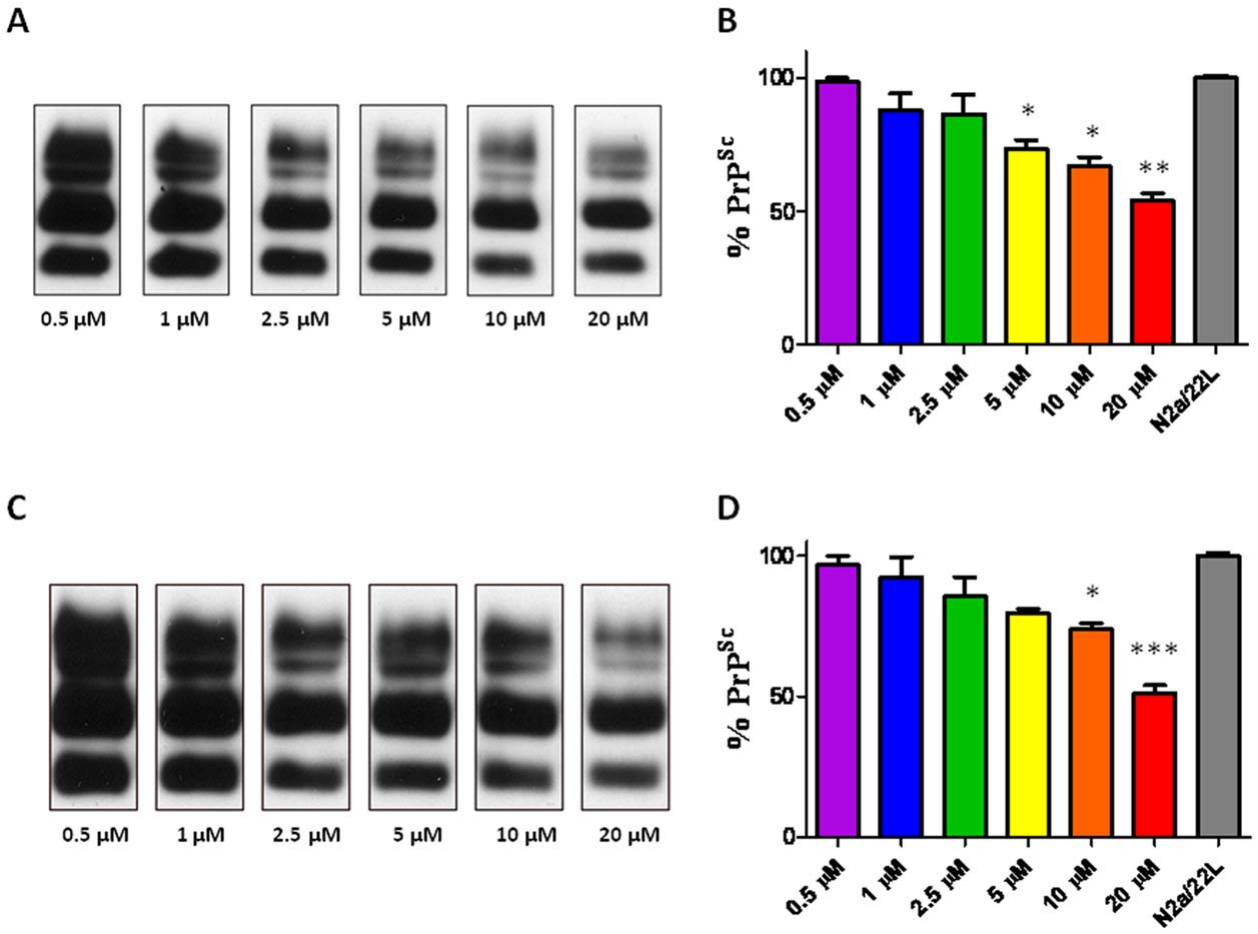
Anti-Prion Activity of Styryl-based Compounds 23I and 59 Styryl-based compounds. A,B) #23I and C,D) #59 were applied to N2a/22L for 72 hrs at 0.5, 1.0, 2.5, 5, 10, and 20 µM. A,C) Blots represent proteinase-K digested cell lysates detected with 6D11 anti-PrP monoclonal antibody. Bands respresent di-, mono-, and non-glycosylated isoforms at approximately 28, 23 and 17 kDa respectively. B,D) Bar graph representations of percent PrP^Sc^ infectivity compared to non-treated control (N2a/22L). B) #23I reduced PrP^Sc^ levels by 98.4%, 88.1%, 86.5%, 73.5%, 67.2% and 56.4%. D) #59 reduced PrP^Sc^ levels by 96.8%, 92.4%, 85.4%, 79.5%, 73.9% and 54.0%. Differences are significant by One-way ANOVA (**p = 0.0011), and *p<0.05 Newman-Keuls *post-hoc* analysis versus N2a/22L as indicated on the graph.

**Figure 2:**
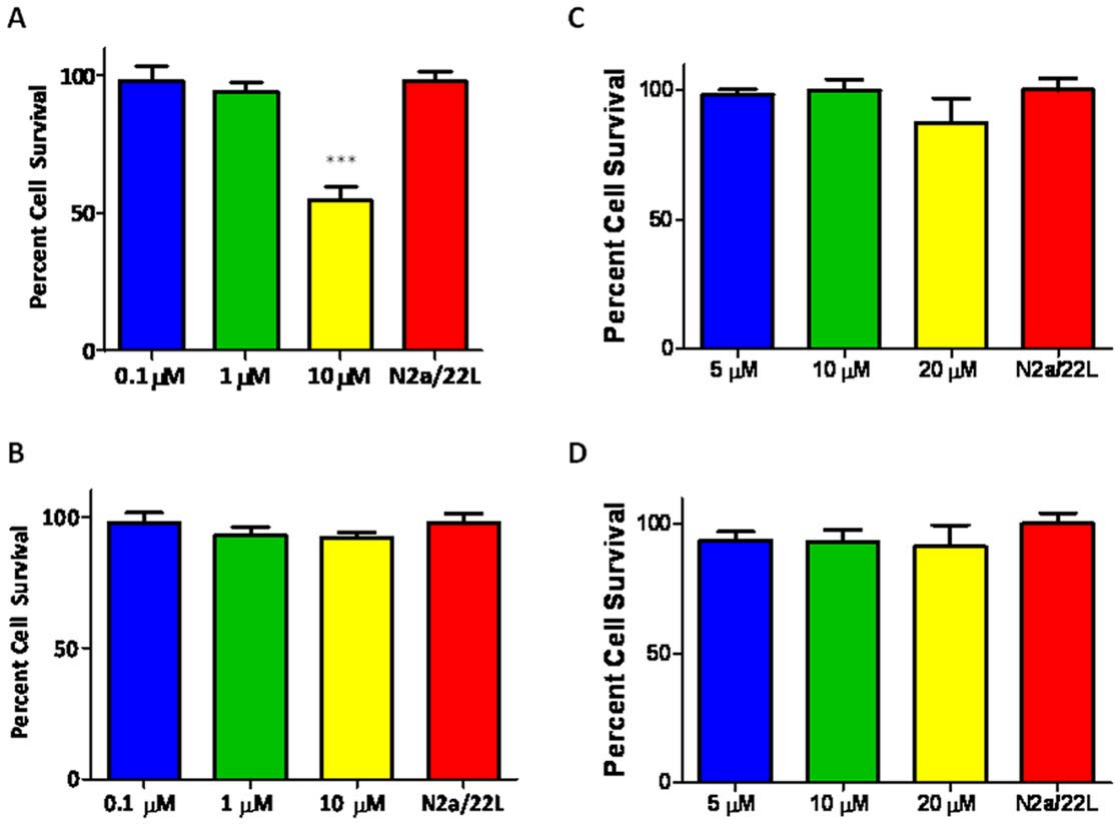
Cytotoxicity Assay. Cytotoxicity of compounds. A) Fluphenazine B) trimipramine C) #23I and D) #59 were applied to N2a/22L cells for 72 hours. Cells were then transferred to 96 well plates and allowed to attach overnight. The MTS colorimetric solution was added and allowed to incubate for 2–3 hours before reading absorbance at 490 nm. Graphs are plotted as percent cell survival compared to non-treated control. Only fluphenazine at 10 µM concentration was significantly toxic; ***p<0.0001, Student's *t*-test, two-tailed.

**Figure 3:**
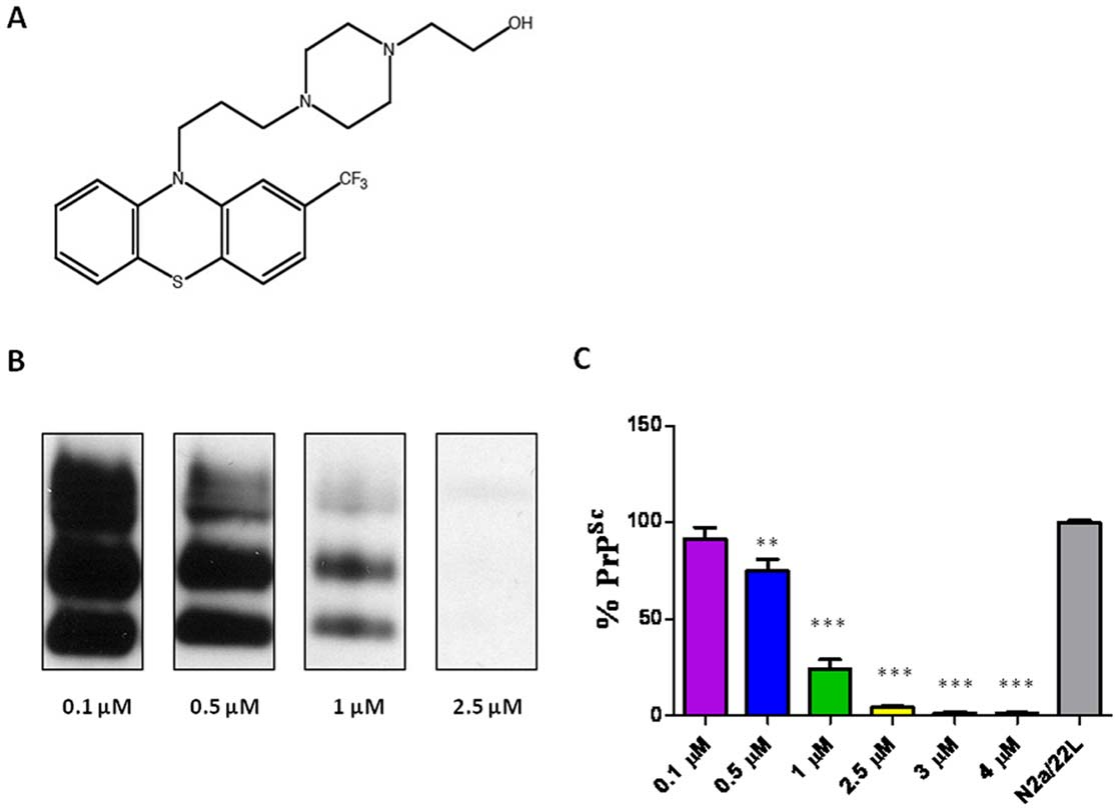
Structure and Anti-Prion Activity of Fluphenazine. Fluphenazine. A) Shows the structure of fluphenazine. Fluphenazine was applied to N2a/22L for 72 hrs at 0.1, 0.5, 1.0 and 2.5 µM. B) Western blot of PK-digested cell lysates. C) Bar graph representation of PrP^Sc^ reduction. Fluphenazine reduces PrP^Sc^ infectivity by 84.8%, 75.5%, 24.0% and 2.9% respectively. Differences are significant by One-way ANOVA (***p<0.0001), and *p<0.05 Newman-Keuls *post-hoc* analysis versus N2a/22L as indicated on the graph.

**Figure 4:**
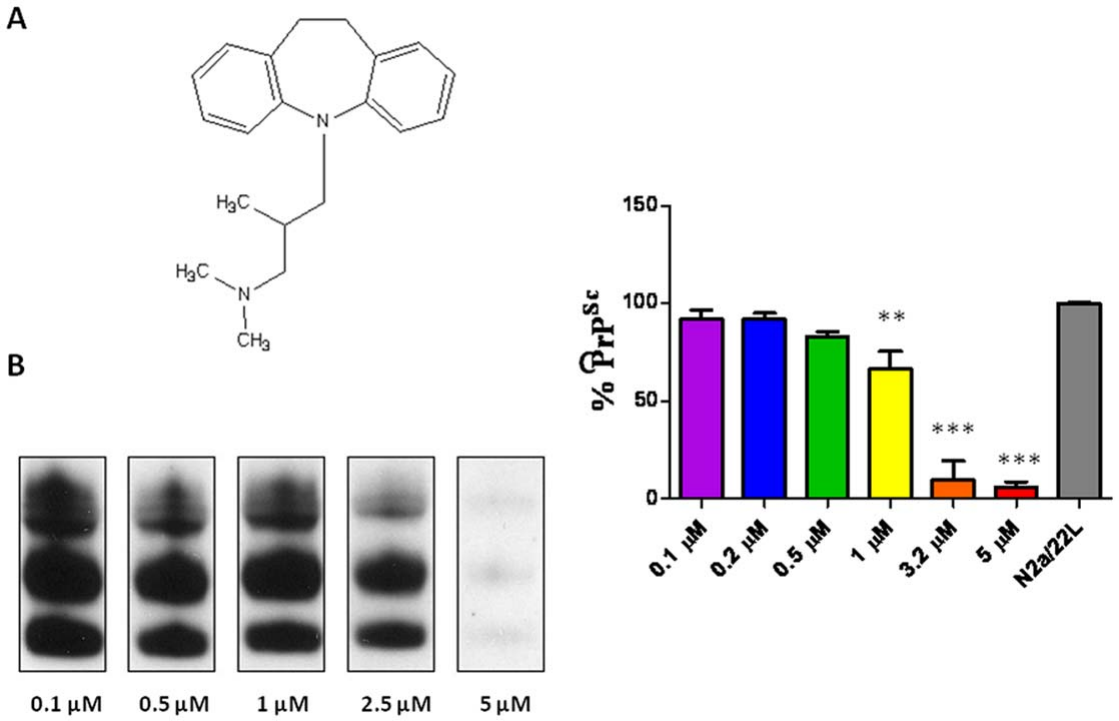
Structure and Anti-Prion Activity of Trimipramine. Trimipramine. A) Shows the structure of trimipramine. Trimipramine was applied to N2a/22L for 72 hrs at 0.1, 0.5, 1.0, 2.5 and 5 µM. B) Western blot of PK-digested cell lysates. C) Bar graph representation of PrP^Sc^ reduction. Trimipramine reduces PrP^Sc^ infectivity by 89.6%, 77.7%, 68.2%, 22.7% and 5.6% respectively. Differences are significant by One-way ANOVA (***p<0.0001), and *p<0.05 Newman-Keuls *post-hoc* analysis versus N2a/22L as indicated on the graph.

**Figure 5:**
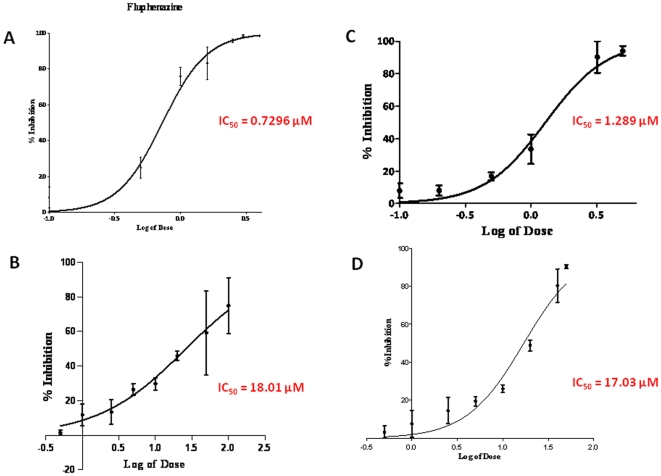
Inhibitory Concentrations (IC_50_) for Fluphenazine, 23I, Trimipramine and 59 Half-maximal inhibitory concentrations (IC_50_) for A) fluphenazine B) #23I C) trimipramine and D) #59. Graphs were plotted as percent inhibition of PrP^Sc^ infectivity versus log of dose. IC_50_ values are 0.7296 µM for fluphenazine, 1.289 µM for trimipramine, 18.01 µM for #23I and 17.03 µM for #59.

N2a/22L cells at passage 5 and above were treated with a library of 68 imaging compounds originally synthesized to bind to and detect amyloid-β fibrils *in vivo* in an initial screen [19]. Of these compounds, only 16, 23I, 26, 27, 52, 59, 63, 67, 69 and 84 showed anti-prion activity. However, only two compounds (#23I and #59) consistently lowered PrP^Sc^ levels in infected cells (Figure 1) with no apparent toxicity as judged by the MTS assay (Figure 2). All the other imaging compounds with anti-prion activity were found to be toxic (data not shown). Fluphenazine and trimipramine were chosen for their tricyclic scaffold and aliphatic side-chain, a structure previously found to be important for anti-prion activity (Figures 3 and 4) [17]. The resulting half maximal inhibitory concentration (IC_50_) for fluphenazine was 0.7296 µM and 1.289 µM for trimipramine. The two imaging compounds had higher IC_50_ values of 18.01 µM for #23I and 17.03 µM for #59 (Figure 5).

### Cytotoxicity Assay

Cytotoxicity, as determined by MTS colorimetric solution, showed no significant cell death in either #23I or #59 at 5, 10 or 20 µM when applied to N2a/22l for 72 hours. Trimipramine was nontoxic at 0.1, 1, and 10 µM. Fluphenazine showed no significant difference in cell viability at 0.1 or 1 µM, but was toxic at a 10 µM concentration. However, the effective anti-prion dose of fluphenazine was much lower with complete abrogation at less than 1 µM (Figure 2).

### Survival of Mice

**Figure 6:**
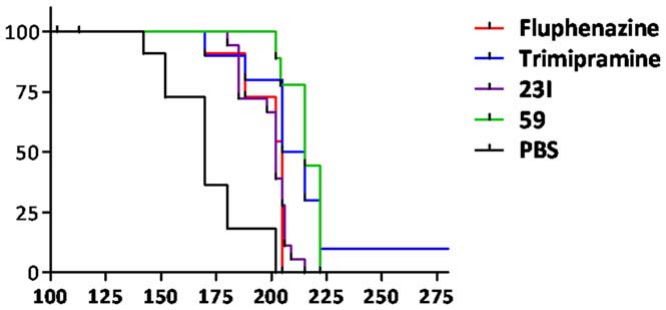
Kaplan-Meier Survival Curve. Survival analysis. The Kaplan-Meier survival curve shows significant treatment effects for all treatment groups. Survival analysis by log-rank test gave *p* values of: *p* = 0.0005 for fluphenazine, *p* = 0.0001 for trimipramine, *p*<0.0001 for #23I, and *p*<0.0001 for #59. The median survival for treatment groups were 205 dpi for fluphenazine, 210 dpi for trimipramine, 202 dpi for #23I, 215 dpi for #59 compared to 170 dpi for control. One animal in the trimipramine group remained clinically asymptomatic at 400 dpi, when it was sacrificed.

Treatment of mice with each compound began immediately following peripheral inoculation with 139A PrP^Sc^. Beginning at 90 days post inoculation with 139A, animals were tested weekly for signs of ataxia, the equivalent of clinical onset and neuroinvasion of prion disease. After 3 weeks of positive scoring by a blinded observer, animals were sacrificed and the date post inoculation of sacrifice recorded and plotted. The Kaplan-Meier survival curve showed significant effects for all treatment groups. Survival analysis by log-rank (Mantel-Cox) test gave a *p* = 0.0005 for fluphenazine, *p* = 0.0001 for trimipramine, *p*<0.0001 for #23I, and *p*<0.0001 for #59 (GraphPad Prism version 5.04). The median survival for treatment groups were 205 dpi for fluphenazine, 210 dpi for trimipramine, 202 dpi for #23I, 215 dpi for #59 compared to 170 dpi for control. This represents a prolongation of the asymptomatic incubation of 21%, 24%, 19% and 26% for fluphenazine, trimipramine, 23I and 59 compared to controls, respectively. One animal from the trimipramine treatment group remained healthy through 400 dpi, showing no signs of cerebellar ataxia (Figure 6).

### PrP^Sc^ in Spleen

**Figure 7:**
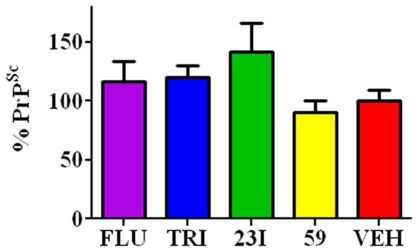
Level of PrP^Sc^ in the Spleen. PrP^Sc^ level in the spleen at 60 dpi. At this time, there is peripheral replication of PrP^Sc^ but CNS invasion has not yet occurred. Spleen homogenates for each group (n = 10) were tested for levels of PrP^Sc^ and plotted with the vehicle level represented as 100% infectivity. Differences between each treated group versus the control group were not significant.

Spleens were homogenized in PBS without Ca^++^ or Mg^++^ and were subjected to sodium phosphotungstic acid precipitation to amplify PrP^Sc^ levels. PrP^Sc^ levels in spleen homogenates averaged 95.8%, 98.6%, 116.3%, 73.8% and 82.1% for fluphenazine, trimipramine, #23I, #59 and vehicle groups respectively (n = 10). Percent PrP^Sc^ was quantified compared to one previous 139A infected animal which was considered 100% infectivity. The difference in PrP^Sc^ levels between groups was not significant by one-way ANOVA (Figure 7).

### PrP^Sc^ in Brain homogenate

**Figure 8:**
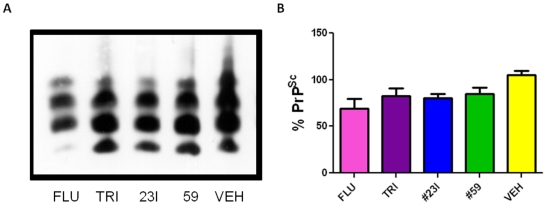
Level of PrP^Sc^ in the Brain. Brain homogenates of clinically symptomatic animals were PK-digested to assess PrP^Sc^ levels. A) Shows a Western blot of a representative single mouse from each treatment group. B) Bar graph representation of total group brain homogenate PrP^Sc^ levels as measured by O.D. of Western blots. The vehicle group is set as 100%. The mean values as a percentage of the vehicle control group are 68%, 83%, 80% and 85% for the fluphenazine, trimipramine, 23I and 59 groups, respectively. (*p*<0.05 by one-way ANOVA). Compared to vehicle control *p* = 0.01 for fluphenazine, *p* = 0.02 for trimipramine, *p* = .002 for 23I and *p* = 0.02 for 59 (by two-tailed t test).

Brain homogenates of all sacrificed animals were subjected to proteinase K digestion and resulting Western blot analysis of PrP^Sc^ showed lower levels of PK-resistant material in brain homogenate for all treatment groups compared to control (p<0.05 by one-way ANOVA) (Figure 8). There was no detectable PrP^Sc^ by Western blot in the brain of the one surviving trimipramine treated mouse sacrificed at day 400 (data not shown).

### Spongiform Density and Astrogliosis

**Figure 9:**
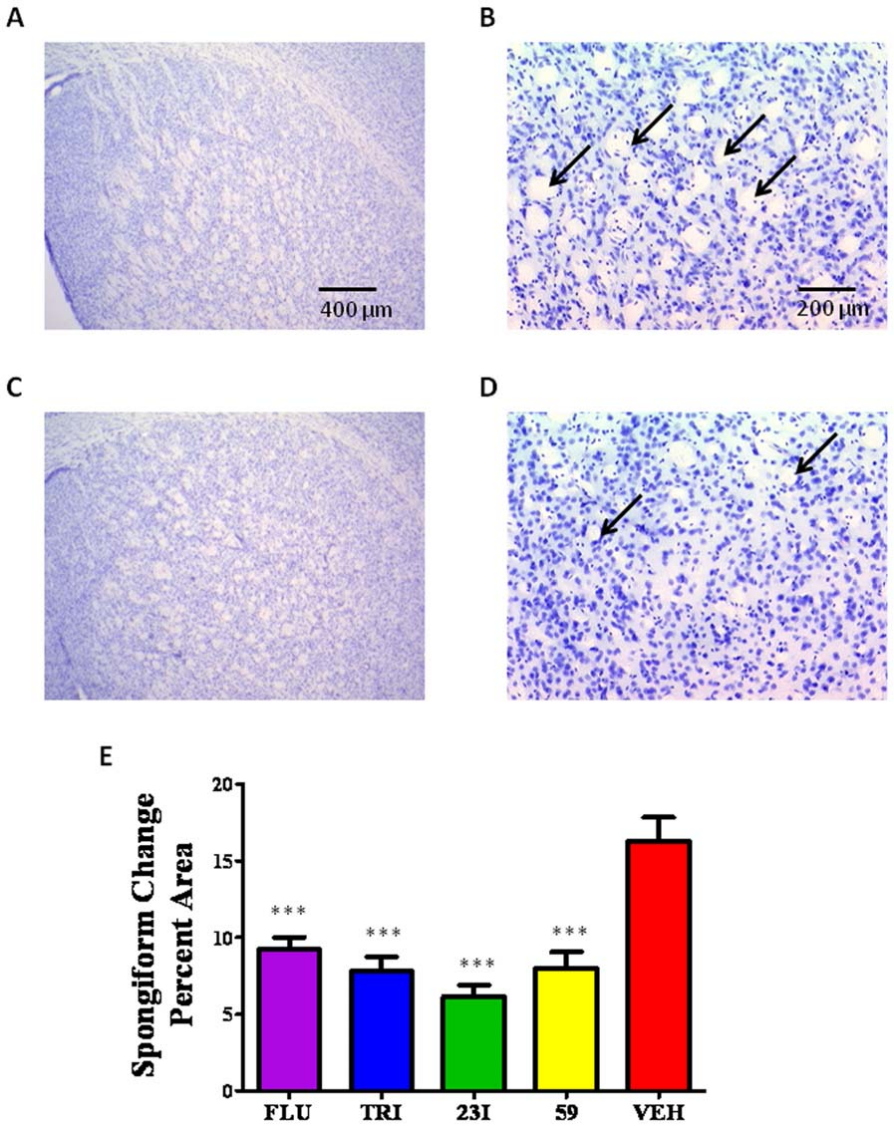
Quantitation of Spongiform Change. Spongiform density. Spongiform change in the basal ganglia of all groups were quantified using Bioquant NovaPrime image analysis. Histological sections were stained with cresyl violet. A,B) Represent a section of basal ganglia of non-treated control animals at 25× and 100× magnification respectively. C,D) Represent a treatment group (fluphenazine) at 25× and 100× magnification. Arrows are highlighting vacuoles representing spongiform change. E) Bar graph representation of spongiform density quantification of all animal groups. Graphs were plotted as percent spongiform change per area. All treatment groups had significantly lower levels of spongiform density compared to non-treated control; ***p<0.0001 by One-way ANOVA; *p<0.05 Newman-Keuls *post-hoc* analysis versus N2a/22L as indicated on the graph.

**Figure 10:**
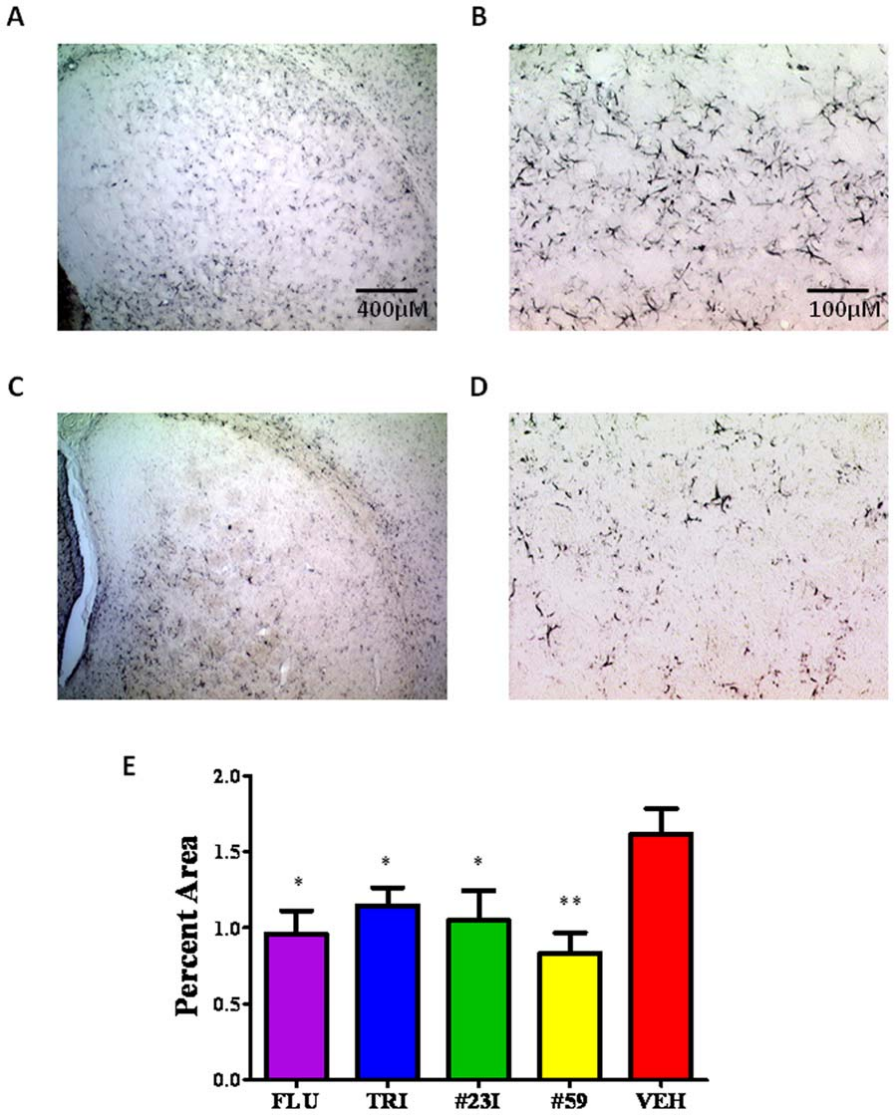
GFAP Immunoreactivity as a Measure of Astrocytosis. Histological sections immunostained with anti-GFAP polyclonal antibody were quantified using Bioquant NovaPrime image analysis. A,B) Histological representation of non-treated control animal at 25× and 100× magnification, respectively (scale bars corresponding to 100 and 400 µm are shown). C,D) Shows representative sections from a treatment group (#23I) at 25× and 100× magnification. E) Shows a bar graph of the immunoreactivity quantitation for all groups. Graphs were plotted as percent immunoreactivity per area. Differences were significant by One-way ANOVA; *p* = 0.0073.

The density of spongiform change and astrogliosis was quantified in the basal ganglia of all animals in tissue stained with cresyl violet and anti-GFAP antibody, respectively. Spongiform change was expressed as the percent area of vacuoles in the fixed sampling grid. The differences in spongiform density was significant for all treatment groups versus control by One-way ANOVA with a Newman-Keuls *post hoc* analysis, *p*<0.0001 (Figure 9). Astrogliosis was quantified as the percent area of GFAP immunoreactive astrocytes in the fixed sampling grid (Figure 10). The differences in GFAP immunoreactivity was significant for all treatment groups versus control by One-way ANOVA, *p* = 0.0073.

### Western Blot Detection and Quantification of PrP^C^

Semiquantitative analysis for areas under the curves representing di-, mono-, and non-glycosylated bands of PrP^C^ were similar in the treated and control mice. Two-way ANOVA analysis showed no significant differences between control mice and trimipramine, fluphenazine, 23I or 59 treated mice for all isoforms of PrP^C^ (data not shown).

### Thioflavin T fluorescence

**Figure 11:**
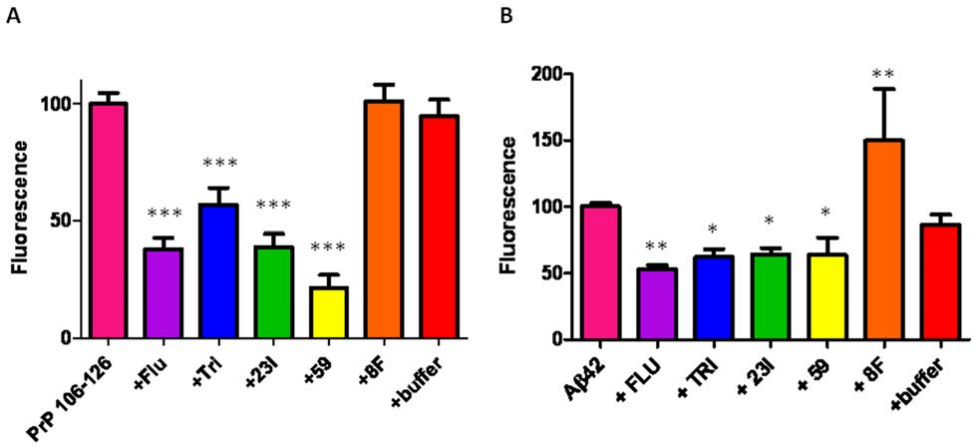
Disaggregation of Pre-formed PrP106-126 and Aβ 42 Fibrils. Thioflavin T fluorescence of compounds incubated with A) PrP 106-126 and B) Aβ42 fibrils for 72 hours. Compounds incubated with both with PrP 106-126 and Aβ42 significantly lowered fluorescence intensity. *p*<0.0001 by One-way ANOVA; *p<0.05, **p<0.01, ***p<0.001 Newman-Keuls *post-hoc* analysis of compounds versus peptide control.

Compounds incubated with aggregated PrP 106-126 or Aβ42 fibrils were measured for fluorescence intensity after 72 hours (Figure 11). Subtracting peptide/Tris as background, fluorescence intensity of compound/peptide/Tris/ThT mixtures were compared to peptide/Tris/ThT as control. Compounds fluphenazine, trimipramine, #23I, and #59 significantly lowered fluorescence intensity in PrP 106-126 aggregates compared to control by Student's *t*-test, two tailed; *p*<0.0001 (Figure 11A). Controls (#8F and buffer) did not show significant differences. Compounds also significantly lowered fluorescence intensity in Aβ42 fibrils; *p*<0.0001 for fluphenazine, trimipramine, and #23I; *p* = 0.0139 for #59; Student's *t*-test, two-tailed (Figure 11B). The buffer control did not show significant difference with Aβ42 fibrils while control 8F showed a significant increase (p<0.01) in fluorescence, indicating it promoted further fibril formation.

## Discussion

We demonstrate the in vivo effectiveness of four compounds for prion infection using 139A PrP^Sc^ in CD-1 wild-type mice. All four compounds were able to significantly prolong the asymptomatic incubation period in peripherally infected mice, as determined by a delay in the presentation of clinical symptoms. In addition, treatment with the compounds reduced the levels of brain PrP^Sc^, as well as, the degree of spongiform change significantly. Hence these compounds not only delayed neuroinvasion but also altered its effectiveness and subsequent severity of brain pathology. Animals were required to be sacrificed three weeks after the onset of clinical symptoms due to humanitarian considerations and IACUC regulations; therefore, we were unable to quantitate whether or not treatment could have prolonged the symptomatic period beyond three weeks. However, we demonstrated that treatment does reduce the severity of disease by showing reductions in the degree of spongiform change and GFAP immunoreactivity, as well as lower levels of PrP^Sc^ in the brains of all the treated groups. These compounds were not toxic in tissue culture using a standard MTS assay with doses used in the current experiment and in the case of fluphenazine and trimipramine utilized clinically. Furthermore, the treated mice showed no signs of toxicity with any of the compounds. The mechanism of action of these compounds is likely, at least in part, related to their ability to hinder the PrP^C^ to PrP^Sc^ interaction. Our evidence for this is that each of the tested compounds is able to disaggregate both Aβ and PrP amyloid fibrils in vitro. A precise understanding of the molecular mechanisms and pathways involved in the PrP^C^ to PrP^Sc^ conversion remains to be elucidated; however, there is abundant evidence of the primal importance of “seeding” by aggregated PrP^Sc^ molecules acting as template for PrP^C^ binding and subsequent conversion to more PrP^Sc^ [22]. This interaction is critically dependent on the correct stereochemistry as supported by the existence of a species barrier for prion infection, related to minor differences in the primary sequence of PrP^C^ in different species. It is not surprising that any compound that may alter or mask the mutual conformational complementarity required in prion propagation will be inhibitory. This likely represents a mechanism of action of these compounds.

We chose to investigate the anti-prion activity of fluphenazine and trimipramine, because of structural similarity to quinacrine, as well as their known good BBB permeability and well characterized clinical safety. Fluphenazine, an antipsychotic, is used in the treatment of schizophrenia, hallucinations, delusions and acute mania. Trimipramine is a tricyclic antidepressant. Quinacrine has been shown to be highly effective at clearing prion infection in tissue culture [17], [23]. However, this result contrasted with in vivo studies where no effect was evident in mouse prion infection models [24], [25]. One study dosed mice peripherally infected with 2% (w/v) of 6PB1 mouse- adapted BSE brain homogenate with 10 mg/kg quinacrine for three weeks. Treated mice showed no clinical improvements or increase in survival time compared to non-treated controls [24]. When quinacrine was tried in humans with Creutzfeldt-Jakob disease, no therapeutic effectiveness was demonstrated, despite the use of high doses [26]. Contrasting with this lack of success of quinacrine treatments *in vivo*, we show both fluphenazine and trimipramine to have good anti-prion therapeutic activity *in vivo* using a mouse model, opening up the possibility of clinical assessments of efficacy.

A number of amyloidophilic compounds have been reported to have anti-prion activity. It was initially shown that Congo red, a standard histological dye used to label amyloid deposits, has anti-prion activity in an in vivo model [27]. However, due to its benzidine structure, Congo red is carcinogenic and toxic, making it unsuitable for animal or human use. We and another group have developed Congo red analogs that have effectiveness in tissue culture, but in vivo effectiveness was much more limited [13], [15]. The Doh-ura group have tested a number of PrP amyloid imaging ligands for anti-prion clearance in cell culture and shown some effectiveness in vivo using Tg20 PrP over-expressing transgenic mice [14], [16]. However, in Tg7 mice and wild type hamsters inoculated with 263K PrP^Sc^, there was no significant prolongation of the incubation period. We describe the effectiveness of two chemically distinct compounds, which were initially developed as amyloid-β imaging agents, as having anti-prion activity both in a cell culture model, as well as, in vivo using wild-type CD-1 mice. These compounds, 59 and 23I, have no toxicity in a standard MTT assay or apparent toxicity in vivo. This raises the possibility that they might serve as lead compounds with anti-prion activity. The mechanism of action of our two compounds, similar to other amyloidophilic agents, is likely the inhibition of the PrP^C^ to PrP^Sc^ interaction, consistent with our finding that both compounds are able to disaggregate pre-formed PrP 106-126 fibrils.

In summary, we demonstrate the in vivo effectiveness of four compounds for peripheral prion infection. Two of these compounds, trimipramine and fluphenazine, are widely used in humans for other indications and are both BBB permeable and very safe. This opens the possibility of clinical evaluation of these compounds for anti-prion activity. We also have identified two amyloidophilic compounds with anti-prion activity from a library originally designed for high affinity binding to Aβ plaques and BBB permeability, which can serve as lead compounds for the design of even more effective drugs. These agents are now being further tested in other in vivo prion models, as well as for therapeutic effectiveness when applied closer to the onset of clinical symptoms.

## Materials and Methods

### Tissue Culture Model of Prion Disease

N2a mouse neuroblastoma cells (ATCC line CCL-131) were maintained in minimal essential medium (MEM) supplemented with heat-inactivated 10% fetal bovine serum, penicillin (100 units/ml) and streptomycin (100 µg/ml) at 37°C in 5% CO_2_. Brains of terminally ill CD-1 mice infected with mouse-adapted 22L prion strain were homogenized (10% w/v) in cold phosphate-buffered saline and 5% sucrose under sterile conditions, as previously described [20], [21]. For infection of N2a cells, the homogenate was further diluted to 2% in Opti-MEM and added to confluent 12.5 cm^2^ flasks (Falcon). After 4–5 hours, an equal volume of regular MEM was added and cells were incubated in the presence of infectious brain homogenate overnight. The cells were then washed twice with PBS and fresh MEM was replaced. Cells were grown until confluence and then split into 1∶4 dilutions and transferred to 25-cm^2^ flasks until the fourth passage when traces of 22L brain homogenate can no longer be detected.

### Treatment of N2a/22L cells with compounds

N2a/22L cells (from the fifth passage after infection and above) were plated in six-well plates. Fluphenazine and trimipramine were applied at concentrations ranging from 0.1–5 µM for 72 hours, while each of the library of 76 amyloid imaging compounds were applied at 1–20 µM for 72 h (see Figure S1 showing the structure of each of the 76 compounds which were screened). A fresh treatment was applied daily until lysis. The level of PK-resistant PrP^Sc^ was measured by Western blot. Each experiment included both a positive control (non-treated N2a/22L cells) and a negative control (non-infected N2a cells). Quinacrine was applied at a concentration of 1.5 µM for 72 h as a treatment positive control. The levels of PrP^Sc^ was expressed as percentages of the average value from a positive control (nontreated N2a/22L cells), while the optic density of the background was taken from negative control lanes (N2a cells). Half maximal inhibitory concentration (IC_50_) values were calculated by plotting percent of PrP^Sc^ reduction versus log of dose.

### Detection and quantification of PrP^Sc^ in N2a/22L cells

Cells were harvested using ice-cold lysis buffer [NaCl, 150 mM; triton X-100, 0.5%; sodium deoxycholate, 0.5%; and Tris-HCl, 50 mM, pH 7.5; with a protease inhibitor cocktail (Roche, Indianaplis, IN, USA)], as previously described [20], [21]. The lysates were centrifuged for 3 minutes at 10,000 g to remove cell debris and the total protein concentration was measured in the supernatant using the bicinchoninic acid assay (BCA; Pierce, Rockford, IL, USA). Aliquots containing 200 µg of total protein were titrated by adding buffer to achieve a final protein concentration of 1 µg/µl. Samples were digested with proteinase K (PK; Roche) for 30 min at 37°C. The enzyme-to-protein weight ratio was 1∶50. PK activity was quenched by adding phenylmethanesulphonyl fluoride to achieve a final concentration of 3 mM. Samples were then centrifuged at 20,000 g for 45 min at 4°C. Pellets were resuspended in PBS and tricine sample buffer (Bio-rad, Hercules, CA, USA) with β-mercaptoethanol (BME), boiled at 95°C for 5 min and then subjected to electrophoresis on 12.5% SDS-polyacrylamide Tris-tricine gels. Following overnight electrophoresis the proteins were transferred onto nitrocellulose membranes (Amersham Biosciences, Piscataway, NJ, USA) for 1 hour at 400 mÅ using CAPS buffer (3-cyclohexylamino-1-propanesulphonic acid) containing 10% methanol. The membranes were blocked with 5% Carnation nonfat milk in TBST (Tris, 10 mM; NaCl, 150 mM; Tween 20, 0.1%, pH 7.5) for 1 h at room temperature and then incubated with anti-PrP Mab 6D11 diluted to 1∶3000 [28]. Following extensive washing in TBST the membranes were incubated with a horseradish-peroxidase conjugated goat anti-mouse antibody (Thermo Scientific, Rockford, IL, USA) and then developed using an enhanced chemiluminescent substrate (ECL Western Blotting Substrate; Pierce). Membranes were applied to autoradiography film (Super RX Fuji Medical XRay Film; Fujifilm, Tokyo, Japan). Developed films were converted into 8-bit grayscale digital files using an Epson Perfection 1200 U scanner (Epson America, Long Beach, CA, USA) and Adobe Photoshop software (Adobe Systems, San Jose, CA, USA) and saved in JPEG format with a resolution of 600 dpi. Quantification of PrP^Sc^ was performed by densitometric analysis using NIH Image J software. Areas under the curves for the three PrP bands representing non-, mono-, and diglycosylated isoforms of the protein were summarized from each sample to calculate total PrP^Sc^ level.

### Cytotoxicity Assay

Compounds used to treat N2a/22L cells were assessed for cytotoxicity by CellTiter 96 AQ_ueoue_ Non-Radioactive Cell Proliferation Assay (Promega, Madison, WI). Treated cells were plated onto 96-well plates in triplicate and allowed to attach overnight. The MTS colorimetric solution [3-(4,5-dimethylthiazol-2-yl)-5-(3-carboxymethoxyphenyl)-2-(4-sulfophenyl)-2H-tetrazolium] was then added and allowed to incubate at 37°C for 2–3 hours. MTS is bioreduced by cells into a formazan product that is soluble in tissue culture medium. The absorbance of the formazan at 490 nm was measured directly from 96-well plates in a Spectra max M2 and using SoftMaxPro software Version 4.8. Viability was determined as percent of control, with control being non-treated cells. Statistical significance of compound toxicity was analyzed by Student's *t*-test, two-tailed (GraphPad Prism, version 5.04; GraphPad Inc., San Diego, CA, USA).

### Treatment in Animal Models

#### Ethics Statement

Animal studies were approved by the NYU School of Medicine Institutional Animal Care and Use Committee (IACUC, protocol 090201-02) and were consistent with the recommendations of the American Veterinary Association.

### Inoculation of CD-1 mice

Female CD-1 mice, aged 2 months, were inoculated by a single intraperitoneal (i.p.) injection of 100 µl of 1% brain homogenate from terminally ill 139A scrapie-infected mice. A single batch of 139A inoculum prepared from a number of pooled brains harvested under sterile conditions was used to inoculate mice.

### Treatment protocol

Fluphenazine-dihydrochloride (Sigma-Aldrich, St. Louis, MO, USA) was intraperitoneally (i.p.) administered daily at 1 mg/kg. Trimipramine maleate salt (Sigma-Aldrich), and the imaging compounds (#23I, #59) were administered i.p. at 10 mg/kg. The doses of fluphenazine and trimipramine were comparable to normal human usage, while doses of the imaging compounds were based on the highest tolerated dose of quinacrine in animal models [17]. Animals were treated immediately after inoculation with 139A brain homogenate, and 5 times a week thereafter. Due to the unknown long-term potential toxic side effects of the imaging compounds, the #23I and #59 groups were treated for 1 month following the 139A challenge while the groups given fluphenazine and trimipramine (which are known to be non-toxic) were continually treated until sacrifice. Treatment groups consisted of 25 mice each with the control group of 25 mice given sterile phosphate-buffered saline. A cohort of 10 animals per treatment and control groups was sacrificed at 60 days to analyze peripheral PrP^Sc^ replication. Beginning at 90 days post inoculation (dpi) and weekly thereafter, the 15 remaining animals per group underwent a test for ataxia in traversing parallel bars.

### Assessment of disease

The parallel bar apparatus contains a series of parallel bars 3 mm in diameter placed 7 mm apart. Animals were scored based on their performance and were sacrificed when scored positive three weeks in a row, indicating early signs of clinical symptoms as previously reported [20], [29]. The observer was blinded to the treatment status of the mice. The earliest detectable clinical symptoms of central nervous system involvement include an impaired activity level and competency when attempting to cross the parallel bars. Symptomatic animals were euthanized with an i.p. injection of ketamine/xylazine 100/10 mg/kg and the clinical diagnosis was confirmed by demonstration of PrP^Sc^ on Western blots from PK-digested brain homogenate. The Kaplan and Meier survival curves of the treated and control animals were analyzed by the log-rank test (GraphPad Prism, version 5.04, GraphPad Software Inc.).

### Analysis of disease

Animals were sacrificed and perfused with heparinized PBS. The tissues collected were the brain, spleen, Peyer's patches and mesenteric lymph nodes. The brain was divided along the longitudinal fissure with the right brain hemisphere (including cerebellum and brain stem) fixed in periodate-lysine-paraformaldehyde (PLP) and the left hemisphere snap-frozen and homogenized for biochemical analysis. Samples designated for biochemical analysis were weighed and homogenized (10% w/v) in Dulbecco's phosphate-buffered saline (DPBS). Homogenates were divided in 150 µl aliquots, flash frozen and stored at −80°C until analysis. Tissue homogenates underwent sodium phosphotungstic acid precipitation to amplify PrP^Sc^ levels in brain and lymphoid tissue. Tissue homogenates were centrifuged at 80× *g* for 1 minute to remove gross cellular debris. Aliquots of the supernatant were mixed with an equal volume of 4% sodium lauroyl sarcosinate (sarkosyl) (Sigma-Aldrich) then incubated for 10 min at 37°C with constant agitation. Benzonase nuclease (Novagen, EMD Chemicals, Gibbstown, NJ, USA) was added at a final concentration of 50 U/ml followed by 1 mM MgCl_2_, then incubation for 30 min at 37°C with constant agitation. Pre-warmed 4% sodium phosphotungstic acid/170 mM MgCl_2_ (pH 7.4) was added and the solution incubated for 30 min at 37°C with constant agitation before centrifugation at 15800× *g* for 30 min. The resulting pellet was resuspended in 0.1% sarkosyl. Samples were then PK-digested as previously described for 1 hour at 37°C. Tricine sample buffer with BME was added and samples boiled at 95°C for 5 min before subjection to electrophoresis on 12.5% SDS-polyacrylamide Tris-tricine gels. Following overnight electrophoresis the protein was transferred onto nitrocellulose membranes and Western Blot for PrP^Sc^ detection as previously described. Immersion-fixed samples were stained with cresyl violet and immunolabeled with anti-PrP mAb 6D11.

### Quantification of Disease

The density of spongiform changes and astrogliosis were quantified using Bioquant NovaPrime v. 6.95 image analysis system (Bioquant, R&M Biometrics Inc., Nashville, TN) on sections stained with cresyl violet and immunostained with polyclonal anti-GFAP antibody (Dako, Denmark), respectively. GFAP (glial fibrillary acidic protein) is the major intermediate filament of astrocytes; it is used as a measure of astrocytosis and for assessing the degree of prion infection as previously reported by us and others [20], [30]. Analysis includes several ROIs in the basal ganglia for spongiform change and astrogliosis. The stereological coordinates of ROI outlines were stored in Bioquant memory with the system randomly superimposing a sampling grid (800×800 µm) over the contours of the traced ROIs. The rectangular test areas (640×480 µm) were placed by the program.

### Western Blot Detection and Quantification of PrP^C^

Brain samples were weighed, homogenized and sonicated (10% w/v) in a buffer containing 20 mM Tris pH 7.5, 250 mM sucrose, 1 mM EDTA, 1 mM EGTA, and Complete® protease inhibitor (Boehringer-Mannheim, Indianapolis IN), as previously described [31]. Samples were centrifuged for 3 min at 10,000× g at 4°C to remove cellular debris. The total protein concentration was assayed by the BCA method. If not used immediately, supernatants were divided into 100 µl aliquots, which were flash frozen and stored at −80°C. Semi-quantitative Western-blot was used to compare the relative content of PrP^C^ among samples containing matched amounts of total protein. Aliquots of brain homogenates containing 20 µg of the total proteins were titrated by adding sample buffer to a final protein concentration of 1 µg/1 µl. Samples were subjected to SDS-PAGE and Western-blotting into nitrocellulose membranes where PrP^C^ was detected with Mab 6D11 (0.05 µg/ml) as described previously [20]. For the densitometric analysis, the exposure time of Western blot membranes was kept standard in all experiments at 30 seconds. Developed films were converted into 8 bit grayscale digital files using a Epson Perfection 4990 scanner (Epson America; Long Beach, CA) and Adobe Photoshop software 7.01 (Adobe Systems; San Jose, CA) and saved in a TIF format with a resolution of 600 dpi. Quantification of PrP^C^ was performed using NIH Image J software v 1.34. Areas under the curves for three PrP^C^ bands representing non-, mono-, and diglycosylated isoforms of the protein were analyzed from each sample.

### Preparation of Aβ42/PrP 106-126 fibrils

Aβ42 was monomerized by treatment with ice-cold 1,1,1,3,3,3-hexafluoro-2-propanol (HFIP) (Sigma-Aldrich) to a final concentration of 1 mM, as previously described [32]. The solution was incubated at room temperature for 60 min, then placed on ice for 5–10 minutes. The solution was then aliquoted to 0.45 mg Aβ42 into non-siliconized microfuge tubes and allowed to evaporate overnight. All traces of HFIP were removed by speedvac if necessary. The dried peptide films were stored at −80°C until resuspension in dimethyl sulfoxide (Sigma-Aldrich) to a final concentration of 5 mM Aβ42. Fibrils were prepared by dilution in 10 mM HCl to a final concentration of 100 µM, vortexed for 15 seconds, followed by 24 hour incubation at 37°C. PrP 106-126 was dissolved in sterile PBS to a final concentration of 1 mM and allowed to spontaneously aggregate at room temperature for 3–5 hours.

### Thioflavin T assay for a destabililzation effect on preformed fibrils

The destabilization effect on preformed fibrils by the compounds was measured by thioflavin T (Sigma-Aldrich) fluorescence, as previously described [32], [33]. Compounds were incubated with either Aβ42 fibrils or aggregated PrP 106-126 at a 1∶2 peptide to compound molar ratio for 1 to 3 days. Controls included the buffer solution for the compounds alone and compound 8F. 8F was chosen at random as a representative compound from among the library of 68 screened imaging compounds which bound to Aβ fibrils but did not show anti-prion activity in tissue culture (see Figure S1 showing a list and structure of all the screened imaging compounds). Thioflavin T was pre-mixed in 50 mM Tris buffer (pH 7.4) to a final concentration of 5 µM then added to peptide/compound complexes. The fluorescence intensity of the peptide/compound/ThT/Tris complexes were measured using an excitation wavelength of 440 nm and an emission wavelength of 482 nm. Readings were taken every 20 seconds over a period of 5 minutes at time points ranging from 0 to 72 hrs. Statistical significance was measured by one-way ANOVA (GraphPad Prism) with a Dunnett post hoc comparison to peptide/ThT/Tris as the reference group.

## Supporting Information

Figure S1 **Names and Structures of the Amyloidophilic Compounds.** Shows the names and structures of the tested 68 amyloidophilic compounds.
(DOC)
