# Supplementary material for: Styryl-Based and Tricyclic Compounds as Potential Anti-Prion Agents
Source: PLoS One. 2011 Sep 13;6(9):e24844. doi: 10.1371/journal.pone.0024844 (PMC3172287; doi:10.1371/journal.pone.0024844)
Supplement: Figure S1 — Names and Structures of the Amyloidophilic Compounds. Shows the names and structures of the tested 68 amyloidophilic compounds. (DOC) [file pone.0024844.s001.doc]

| Label | Structure |
| --- | --- |
| 2 |  |
| 4 |  |
| 6 |  |
| 7 |  |
| 8 |  |
| 8F |  |
| 8H |  |
| 9 |  |
| 10 |  |
| 11 |  |
| 12 |  |
| 16 |  |
| 17 |  |
| 18 |  |
| 19 |  |
| 20 |  |
| 21 |  |
| 22 |  |
| 23 |  |
| 23B |  |
| 23I |  |
| 24 |  |
| 26 |  |
| 27 |  |
| 51 |  |
| 52 |  |
| 53 |  |
| 54 |  |
| 55 |  |
| 56 |  |
| 57 |  |
| 58 |  |
| 59 |  |
| 60 |  |
| 61 |  |
| 63 |  |
| 64 |  |
| 66 |  |
| 67 |  |
| 68 |  |
| 69 |  |
| 70 |  |
| 71 |  |
| 72 |  |
| 73 |  |
| 74 |  |
| 75 |  |
| 76 |  |
| 77 |  |
| 78 |  |
| 80 |  |
| 82 |  |
| 83 |  |
| 84 |  |
| 85 |  |
| 86 |  |
| 87 |  |
| 88 |  |
| 89 |  |
| 90 |  |
| 91 |  |
| 92 |  |
| 94 |  |
| 95 |  |
| 96 |  |
| 97 |  |
| 98 |  |
| 100 |  |
